# Antivirals that target the host IMPα/β1-virus interface

**DOI:** 10.1042/BST20200568

**Published:** 2021-01-13

**Authors:** Alexander J. Martin, David A. Jans

**Affiliations:** Nuclear Signaling Lab., Department of Biochemistry and Molecular Biology, Biomedicine Discovery Institute, Monash University, Melbourne, Australia

**Keywords:** antiviral agents, dengue virus, importin, ivermectin, SARS-CoV-2, Zika virus

## Abstract

Although transport into the nucleus mediated by the importin (IMP) α/β1-heterodimer is central to viral infection, small molecule inhibitors of IMPα/β1-dependent nuclear import have only been described and shown to have antiviral activity in the last decade. Their robust antiviral activity is due to the strong reliance of many different viruses, including RNA viruses such as human immunodeficiency virus-1 (HIV-1), dengue (DENV), and Zika (ZIKV), on the IMPα/β1-virus interface. High-throughput compound screens have identified many agents that specifically target this interface. Of these, agents targeting IMPα/β1 directly include the FDA-approved macrocyclic lactone ivermectin, which has documented broad-spectrum activity against a whole range of viruses, including HIV-1, DENV1–4, ZIKV, West Nile virus (WNV), Venezuelan equine encephalitis virus, chikungunya, and most recently, SARS-CoV-2 (COVID-19). Ivermectin has thus far been tested in Phase III human clinical trials for DENV, while there are currently close to 80 trials in progress worldwide for SARS-CoV-2; preliminary results for randomised clinical trials (RCTs) as well as observational/retrospective studies are consistent with ivermectin affording clinical benefit. Agents that target the viral component of the IMPα/β1-virus interface include N-(4-hydroxyphenyl) retinamide (4-HPR), which specifically targets DENV/ZIKV/WNV non-structural protein 5 (NS5). 4-HPR has been shown to be a potent inhibitor of infection by DENV1–4, including in an antibody-dependent enhanced animal challenge model, as well as ZIKV, with Phase II clinical challenge trials planned. The results from rigorous RCTs will help determine the therapeutic potential of the IMPα/β1-virus interface as a target for antiviral development.

## Introduction

Compartmentalisation is a key feature of the eukaryotic cell, with transport between the different compartments essential for cellular function [1]. The double membrane of the nuclear envelope encircles and thereby defines the nuclear compartment, which sequesters the cell's genetic material, as well as the processes of RNA transcription and subsequent RNA maturation processes such as mRNA splicing. Protein translation occurs in the cytoplasmic compartment, so that specific signal-dependent transport into and out of the nucleus is necessary for the cell to function; i.e. mRNA needs to be exported to the cytoplasm for translation to occur, while all components of the protein machinery that mediate and/or regulate key nuclear processes such as transcription [2–4] and mRNA splicing/processing need to be imported into the nucleus.

The transport of proteins >45 kDa into and out of the nucleus is mediated by members of the importin (IMP) superfamily, of which there are multiple distinct α and β forms [5,6]. The best-characterised pathway by which host proteins enter the nucleus is that mediated by the IMPα/β1 heterodimeric transport complex [7]. Host proteins dependent on IMPα/β1 for nuclear import include those central to the antiviral response, such as members of the signal transducer and activators of transcription (STATs) and nuclear factor κ-light-chain-enhancer of activated B cells (NF-κB) transcription factor families.

Many viruses exploit the host cell nuclear trafficking machinery, and in particular, the pathway mediated by the host IMPα/β1 heterodimer, in order to enhance their ability to replicate or to subvert the host immune response [3]. Nuclear transport of particular viral proteins has been shown to be critically important for infection by flaviviruses (e.g. dengue virus — DENV or Zika virus — ZIKV; non-structural protein 5 — NS5), lentiviruses (e.g. human immunodeficiency virus — HIV-1; integrase — IN), alphaviruses (e.g. Venezuelan equine encephalitis virus — VEEV; capsid protein — CP), alphainfluenza viruses (Influenza A; nucleoprotein — NP), lyssaviruses (e.g. P1 and P3 forms of the Rabies virus — RV; phospho — P — protein), orthopneumoviruses (e.g. respiratory syncytial virus — RSV; matrix — M — protein), and others [3,4,8–13]. Over the last decade or so, studies using high-throughput screening (HTS) approaches targeting the host–virus interface (i.e. IMPα/β1 recognition of viral proteins) have identified small molecular inhibitors [14–17], which target either the host IMPα/β1 heterodimer, or the specific viral protein [18]. Many these are exciting prospects as antiviral drugs.

## The host cell nuclear import pathway

**Figure 1.**
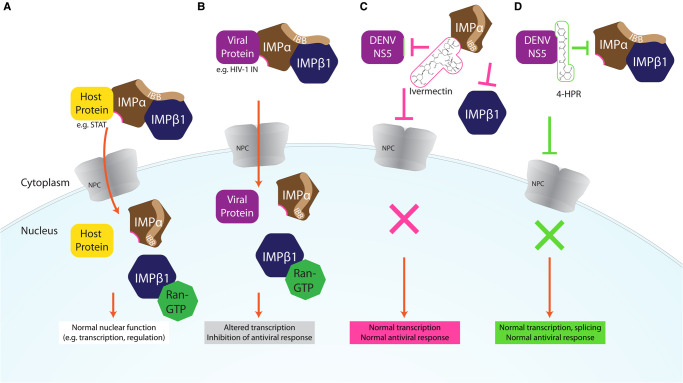
Schematic representation of the host IMPα/β1-dependent nuclear import pathway (**A**), showing how it is co-opted by viral proteins in viral infection (**B**), and how small molecule antivirals can impact the pathway (**C**,**D**). (**A**) Host proteins (e.g. transcription factor STAT-2) contain nuclear localisation signals which are recognised by IMPα (brown), after the autoinhibitory IMPβ-binding (IBB) domain of IMPα binds IMPβ1, forming the IMPα/β heterodimer. This complex is then translocated across the nuclear pore complex (NPC), and the cargo is released after the binding of Ran-GTP to IMPβ1 dissociates the complex. The cargo can then carry out its normal nuclear function, such as transcriptional regulation of the antiviral response. (**B**) During viral infection, specific NLS-containing viral proteins (e.g. HIV-1 IN, purple) are imported into the nucleus by the same IMPα/β1 dependent mechanism, where they can interfere with normal cellular functions, such as altering transcription to antagonise the antiviral response in order to maximise the rate of virus production. (**C**) Ivermectin (pink) binds IMPα, dissociating the IMPα/β1 heterodimer and preventing binding to its viral (as well as host) protein target(s), thereby preventing its nuclear import and downstream transcriptional effects. (**D**) 4-HPR (green) specifically binds viral protein DENV2 NS5, preventing binding of the IMPα/β1 and nuclear import, and associated downstream effects on transcription and splicing.

**Figure 2.**
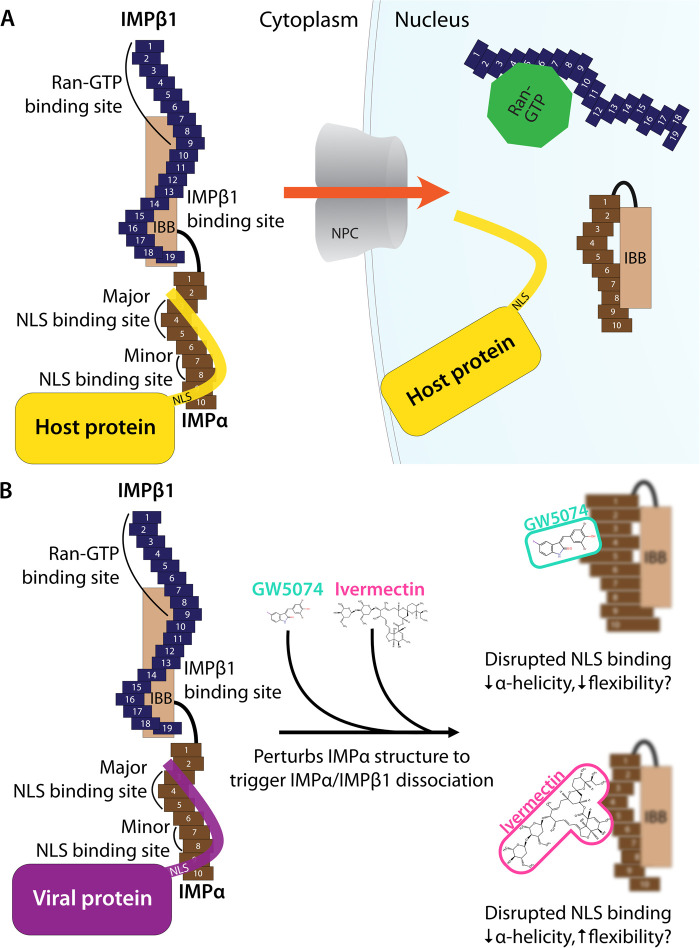
Schematic representation of IMPα and IMPβ1 structural/functional domains; impact of inhibitors. (**A**) Schematic representation of the IMPα/β1 complex based on the crystal structures of human IMPα and IMPβ1. IMPα comprises 10 armadillo (ARM) repeats and an IMPβ-binding (IBB) domain. IMPα ARM repeats 2–4 form the major cargo NLS binding site, while ARM 7–8 form the minor NLS binding site. IMPβ1 comprises 19 HEAT repeats, where repeats 7–19 bind the IMPα IBB domain, and Ran-GTP binds to the N-terminus/repeat 8. In the cytoplasm, IMPβ1 (blue) binds IMPα (brown) via the IBB domain, exposing the major NLS binding site for cargo protein (yellow) binding. After transport into the nucleus across the NPC, Ran-GTP binds to IMPβ1, altering its conformation and displacing and releasing the IBB domain, which then binds the NLS binding sites in an autoinhibitory manner, preventing NLS binding. (**B**) Ivermectin and GW5074 bind directly to IMPα to perturb its structural integrity as confirmed by biophysical measurements including circular dichroism, thermostability analysis, and analytical ultracentrifugation [44,47], to reduce IMPα-helicity/impact flexibility, and thereby prevent IMPα binding to NLS-containing proteins or IMPβ1.

To gain access to the nucleus, proteins generally require nuclear localisation signals (NLSs), which are recognised by either the IMPα subunit within the IMPα/β1 heterodimer, or by one of the IMPβs directly [5,6,2,5]. The IMP:cargo complex is then translocated through the nuclear pore complex (NPC). Once within the nucleus, binding of Ran-GTP to IMPβ dissociates the complex, freeing the protein cargo to perform its nuclear function [19,20]. This process is illustrated in Figure 1. As hinted at above, there are multiple distinct IMPαs; these can be grouped into three subfamilies, but all are highly homologous, and function in the same way within the IMPα/β1 heterodimer to mediate nuclear import through a shared domain structure of ten α-helical armadillo (ARM) repeats involved in NLS recognition, and a flexible N-terminal importin-β-binding (IBB) domain (see Figure 2) [21]. X-ray crystallographic evidence indicates that NLS-binding is most commonly mediated by IMPα ARM repeats 2–4 (‘major NLS-binding site’) [22], with IMPα ARM repeats 6–8 (‘minor NLS-binding site’) [23] used to a lesser extent, as well as in combination with the major site in the case of certain NLSs. The IBB domain itself contains an NLS-like sequence, so that when the IMPα IBB domain is not bound to IMPβ1, this sequence is bound in the NLS-binding pocket [24], essentially ‘autoinhibiting’ IMPα to prevent binding to NLS-containing cargoes in the absence of IMPβ1 [20,21]. The distinct IMPαs are believed to have certain differences in NLS-binding specificity, as well as tissue distribution [3,21,25,26], potentially having more specialised roles in transporting particular import cargoes and not others [2]. STAT1, for example, has been reported to interact with IMPα5 and not IMPα1 [27], while the Ran nucleotide exchange factor RCC1 appears to be transported into the nucleus only by IMPα3/α4 [28]. Consistent with this, certain viral proteins have been identified as being recognised only by specific IMPαs; these include Hendra and Nipah virus W protein [25,29] and avian virus Influenza A nucleoprotein (NP) which preferentially bind IMPα3, whereas mammalian virus Influenza A NP binds IMPα7 [30].

## Viral proteins subvert the host nuclear import machinery

The host cell nuclear transport can be exploited by viral proteins that target nuclear processes to antagonise the host antiviral response. These include DENV NS5, which accesses the nucleus in order to impact many host nuclear processes (Figure 1); this leads to a range of outcomes such as delay of production of interleukin 8 (IL-8) that favour a host cell environment conducive to viral replication [8,31], effects on host cell spliceosome complexes to reduce the efficiency of pre-mRNA splicing of genes important for the antiviral response [32], and inhibition of recruitment of Paf1/RNA polymerase II complex component PAF1C to suppress interferon (IFN)-stimulated genes [33]. Other nuclear localising viral proteins that antagonise the host antiviral response, include RV P3-protein, in part though binding to STAT-1 and promyelocytic leukaemia tumour suppressor protein to impact IFN signalling [34], and RSV M, which modulates infected host cell transcriptional outcomes [3,35].

In the case of other proteins such as HIV-1 IN and influenza A NP, nuclear access is critical to the viral life cycle [4]. Dependent on IMPα/β1 [36], for example, nuclear import of HIV-1 IN/the HIV-1 preintegration complex (PIC) is essential to enable the DNA form of the HIV-1 genome to be integrated into the host DNA to enable productive infection [4,9,36,37]. VEEV CP, in contrast, appears to bind to the host IMPα/β1 heterodimer, as well as the IMPβ-homologue nuclear export protein EXP1, to form a tetrameric complex that accumulates at the nuclear pore and blocks nuclear import of host proteins, thereby reducing the host IFN-α/β response [38,39]. In the case of SARS-CoV-1, the causative agent of severe acute respiratory syndrome (SARS), open reading frame (ORF) 6 has been shown to target host IMPα, and sequester it at the rough ER/Golgi, to prevent its key role in mediating STAT-1 nuclear import, thus mollifying the host cell antiviral response [40].

The critical importance of the IMPα/β1-virus axis to viral infection has been formally demonstrated for many viruses, including DENV, where mutagenesis of the NS5 NLS nuclear targeting through impaired IMPα/β1 recognition results in an attenuated virus and markedly reduced infectious virus production [8]. In addition, several structurally distinct small molecule inhibitors that inhibit NS5 nuclear import (see below), also reduce infectious virus production, underlining the critical importance of nuclear targeting of NS5 in DENV infection. Similar results have been obtained for HIV, where inhibiting the nuclear import of HIV-1 IN protein dramatically reduces HIV-1 infection [41,42], and for VEEV, where mutations within the CP NLS region result in attenuated nuclear accumulation, and are correlated with a non-pathogenic phenotype [39]. Finally, that nuclear trafficking of RV P-protein is critical to infection is implied by the observation that an attenuated non-lethal chicken embryo (CE) cell-adapted strain (Ni-CE) of the highly pathogenic RV Nishigahara (Ni) strain, harbours mutations that impact nucleocytoplasmic distribution [12].

### Inhibitors targeting IMPα/β1: ivermectin

HTS using chemical compound libraries and recombinant proteins has been used to identify many small molecule inhibitors targeting host IMPα/β (see Table 2). Ivermectin (22,23-dihydroavermectin B), the best-studied of these, is a macrocyclic lactone produced by the bacterium *Streptomyces avermitilis*, which was discovered in 1975, and approved for use in humans as an antiparasitic agent 10 years later. It is US FDA-approved for treating a range of ectoparasitic and endoparasitic infestations, with an excellent safety profile and millions of people treated globally for onchocerciasis (river blindness) and lymphatic filariasis [43]. Ivermectin is one of many agents listed on the WHO List of Essential Medicines, with the work identifying it/establishing its properties recognised by the Nobel Prize for Physiology or Medicine in 2015.

**Table 1.** *In vitro* properties of IMPα inhibitors with antiviral effects

Compound	Documented action in nuclear import (IC50)	Antiviral against	Effective concentration (assay)/fold reduction (assay)
Ivermectin^1^ 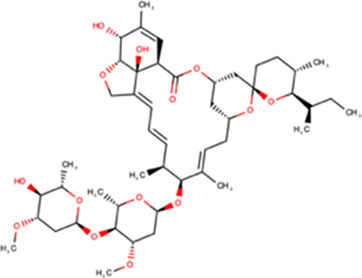	Inhibits interaction *in vitro* of IMPα with HIV IN [14], DENV2 NS5 (1 μM) [42,44], T-ag [15], Hendra V (15 μM) [48], IMPβ1 (7 μM) [44] Inhibits interaction of IMPα with T-ag and NS5 in a cell context as visualised by quantitative BiFc [44] Inhibits CoIP from cell lysates of IMPα with T-ag, Adenovirus EIA [81] Inhibits nuclear accumulation in transfected cells of IMPα/β1- but not β1-recognised viral proteins such as T-ag [16,42], DENV2 NS5 [42], VEEV CP [16], PRV UL42 [60], HIV-1 IN [14,42], hCMV UL44 [42], Influenza A vRNPs [11] as well as host cargoes (e.g. [8,42,46]) Reduces nuclear localisation in infected cells of DENV1-4 NS5 [82], VEEV CP [51] and adenovirus E1A [81]	Betacoronavirus SARS-CoV-2	EC_50_ = 2.2/2.8 μM (qPCR/ released/cell-associated virus) [59] 5 μM > 5000-fold [59]
		HIV-1 (VSV-G-pseudotyped NL4-3.Luc.R-E-HIV)	50 μM > 2-fold (luciferase) [42]
		Influenza VLPs (avian influenza A/MxA escape mutants)	10 μM total inhibition (luciferase) [11]
		Flaviviruses YFV (17D)	EC_50_ = 5/0.5 nM (CPE/qPCR) [83] 3 μM > 50000-fold (PFU) [46]
		DENV1 (EDEN1)	EC_50_ = 2.3/3.0 μM (CFI, 2 hosts) [82]
		DENV2 (NGC)	EC_50_ = 0.7 μM (qPCR) [83] EC_50_ = 3.3/0.4 μM (PFU, 2 hosts^2^) [44]
		DENV2 (EDEN2)	EC_50_ = 0.4/0.6 μM (pfu/qPCR) [44] EC_50_ = 2.1/1.7 μM (CFI, 2 hosts) [82] 50 μM total inhibition (PFU) [42]
		DENV3 (EDEN3)	EC_50_ = 1.7 μM (CFI) [82]
		DENV4 (EDEN4)	EC_50_ = 1.9 μM (CFI) [82]
		WNV (NY99)	EC_50_ = 4 μM (qPCR) [83]
		WNV (MRM61C)	EC_50_ = 1/0.5 μM (PFU/qPCR) [44]
		ZIKV (Asian/Cook Islands/ 2014)	EC_50_ = 1.6/1.3 μM (PFU, 2 hosts^2^) [44]
		Alphaviruses Chikungunya virus (CHIKV-Rluc) Sindbis (HR) Semliki forest virus VEEV (TC83)	EC_50_ = 1.9/0.6 μM (luciferase, 2 hosts) [46] 3 μM > 5000-fold (PFU) [46] 3 μM > 200-fold (PFU) [46] 1 μM c. 20-fold (PFU) [51]
		Hendra (Australia/ Horse/1994)	est. EC_50_ = 2 μM (TCID/luciferase) [48]
		Adenovirus HAdV-C5 HAdV-B3	EC_50_ = c. 2.5 μM; 10 μM 20-fold (qPCR) [81] 10 μM c. 8-fold (qPCR) [81]
		BK polyomavirus (BKPyV)	Est. EC_50_ 1.5 μM (PFU/CPE/qPCR) [84]
		Pseudorabies	Est. EC_50_ c. 0.8 μM 1000-fold [60]
Gossypol^3^ 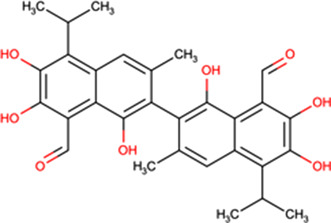	Inhibits interaction *in vitro* of IMPα with Hendra Virus V (10 μM) [48] Inhibits nuclear accumulation in WNV infected cells of NS5 [49]	WNV (MRM61C)	10 μM 100-fold (PFU) [49]
		Hendra (Australia/ Horse/1994)	10 μM 6-fold (TCID/luciferase) [48]
GW5074 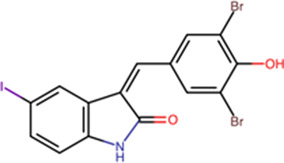	Inhibits interaction *in vitro* of IMPα with DENV2 NS5 (5 μM) [47], Hendra (V 15 uM) [48], IMPβ1 (10 μM) [47] Inhibits nuclear accumulation in DENV2 infected cells of NS5 [47]	Flaviviruses DENV2 NGC	EC_50_ = 0.8/1.4 (PFU/PCR) [47]
		ZIKV (Asian/Cook Islands/ 2014)	EC_50_ = 0.3/0.5 (PFU/PCR) [47]
		WNV (MRM61C)	EC_50_ = 5.2/4.8 (PFU/PCR) [47]
Otava 1111684 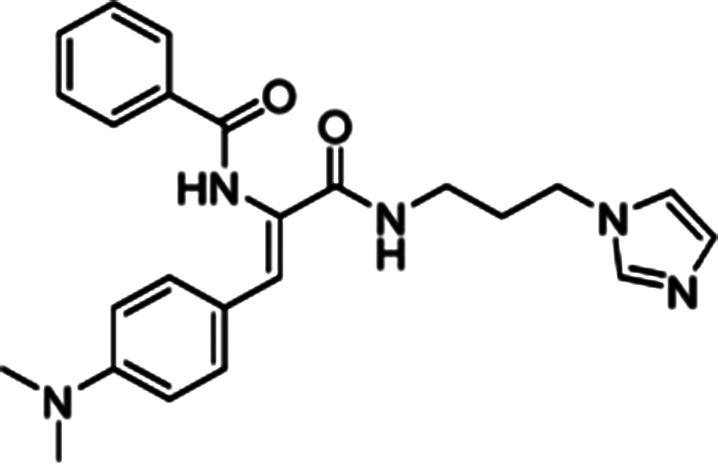	Inhibits interaction *in vitro* of IMPα with VEEV CP [17] Inhibits nuclear accumulation of VEEV CP-GFP in transfected cells [17] Reduces nuclear localisation in infected cells of VEEV CP [17] Reduces rate/extent of VEEV CP-GFP nuclear import (FRAP) [17]	Alphavirus VEEV (TC83)	EC_50_ = 9.9 μM (lum)^4^ [17] 10 μM > 100-fold [17]

1 US Food and Drug Administration (FDA)-approved broad-spectrum antiparasitic agent, including against endoparasitic infestations (strongyloidiasis, onchocerciasis) and ectoparasites causing scabies, pediculosis and rosacea [66]; reported to inhibit helicase activity of DENV2/YFV/WNV NS3 in a FRET-based assay [83];

2 Includes *ex vivo* model of human infection in PBMCs;

3 Abandoned as human male contraceptive due to side effects (hypokalaemic paralysis, testicular damage) [85,86];

4 Selectivity index of 3.7.

**Abbreviations**: ADE, antibody-dependent enhancement of infection; BiFc, bimolecular fluorescence complementation; DENV, Dengue virus; CFI, cell-based flavivirus infection assay (immunostaining for virus); CoIP, co-immunoprecipitation; CP, capsid protein; CPE, cytopathogenic effect (host cell); CHIKV, Chikungunya virus; EC50, half-maximal effective concentration; Est., estimated; FRAP, fluorescence recovery after photobleaching; FRET, fluorescence resonance energy transfer; GFP, green fluorescent protein; HAdV, human adenovirus; hCMV, human cytomegalovirus; HCV, hepatitis C virus; HIV, human immunodeficiency virus; IBB, importin β-binding domain; IC50, half-maximal inhibitory concentration; IN, integrase; lum, luminescence assay; NS5, non-structural protein 5; PCR, polymerase chain reaction; PFU, plaque forming units (infectious virus); PRV, pseudorabies virus; RdRp, RNA-dependent RNA polymerase; SV40, simian virus 40; T-ag, SV40 large T-antigen; TCID, tissue culture infectious dose (estimation of viral load based on CPE); VEEV, Venezuelan equine encephalitis virus; VLP, virus-like particle; vRNP, viral ribonucleoprotein complex; VSV-G, vesicular stomatitis virus glycoprotein; WNV, West Nile virus; YFV, Yellow Fever virus; ZIKV, Zika virus.

**Table 2.** In vitro properties of viral protein cargo-targeted inhibitors with antiviral effects

Compound	Documented action (IC50)	Antiviral against	Effective concentration (assay, host)/fold reduction in virus production (assay, host cell line)
Mifepristone^1^ 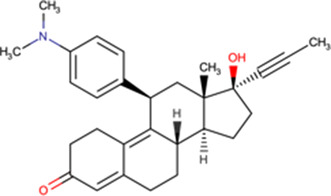	Inhibits interaction *in vitro* of HIV IN with Impα/β (27 μM) [14] Binds to HIV IN core domain but not Impα/β (HSQC NMR) [37] 50 μM inhibits GFP-HIV-1 IN nuclear accumulation in transfected cells [14] 5 μM reduces HIV-1 IN nuclear accumulation > 5-fold in *in vitro* reconstituted nuclear transport assay [37] 5 μM reduces VEEV CP nuclear accumulation in infected cells [51] Reduces rate/extent of VEEV CP-GFP nuclear import (FRAP) [52] 50 μM reduces HAdV genome nuclear import (cellular fractionation/qPCR) [53]	HIV-1	10 μM > 8-fold (EGFP reporter virus, CEMx174) [50] 10 μM >20-fold (ELISA, PBMC) [50] 200 μM > 3-fold (luciferase, HeLa) [42]
		VEEV	EC50 = 19.9 μM (PFU, Vero) [52] 10 μM > 10-fold (PFU, U87MG) [51] 10 μM > 5-fold (PFU, Vero) [51] 10 μM > 15-fold (PFU, Vero) [52]
		Human adenovirus HAdV5	EC50 = 2 μM (PFU) [53]
Mifepristone analogue 50^2^ 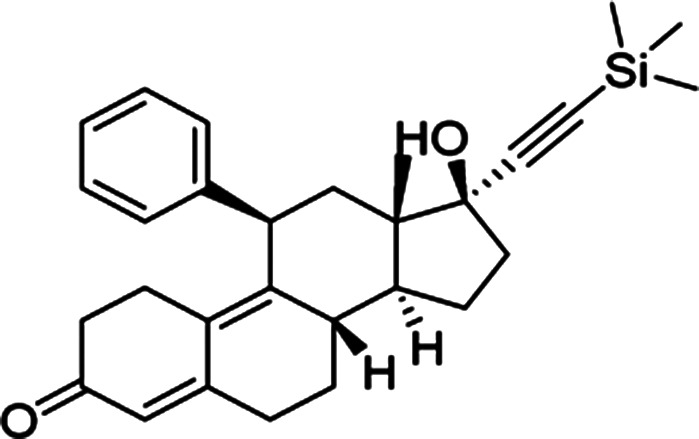	50 μM inhibits nuclear import of transfected GFP-CP (FRAP) Reduces recovery of VEEV CP-GFP nuclear fluorescence after FRAP [52] 50 μM inhibits nuclear accumulation of CP in infected cells [52]	VEEV	EC50 = 7.2 μM (luciferase, Vero) [52] 10 μM > 7-fold (PFU, Vero) [52]
Budesonide^3^ 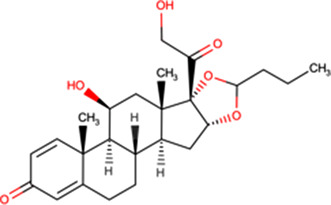	Inhibits interaction *in vitro* of HIV IN with Impα/β (1.2 μM) [37] Binds to HIV IN core domain but not Impα/β (HSQC NMR) [37] 5 μM reduces HIV-1 IN nuclear accumulation > 2-fold in *in vitro* reconstituted nuclear transport assay [37] 100 μM inhibits HIV PIC nuclear import [37]	HIV-1	EC50 = 79.4 μM (luciferase, MT-2, single cycle) [37] EC50 = 49.4 μM (luciferase, TZMbl) [37]
Flunisolide^4^ 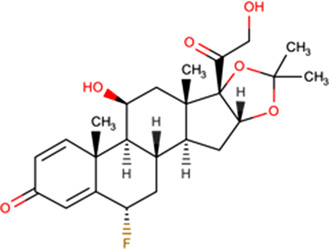	Inhibits interaction *in vitro* of HIV IN with Impα/β (1.4 μM) [37] Binds to HIV IN core domain but not Impα/β (HSQC NMR) [37] 250 μM inhibits HIV PIC nuclear import [37]	HIV-1	EC50 = 77.5 μM (luciferase, MT-2, single cycle) [37] EC50 = 105 μM (luciferase, TZMbl) [37]
G281-1564 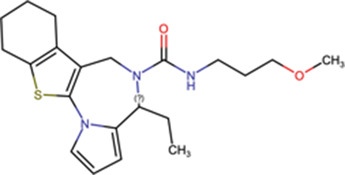	Inhibits interaction *in vitro* of VEEV CP and Impα/β (12 μM) [16] 50 μM inhibits extent and rate of GFP-CP nuclear accumulation in transfected cells (1.8-fold, 1.4-fold, respectively) [16]	VEEV	EC50 = 10.8 μM (luciferase, Vero)^5^ [16] 50 μM > 10-fold (PFU, Vero) [16]
N-(4-hydroxyphenyl) retinamide/fenretinide/4-HPR^6^ 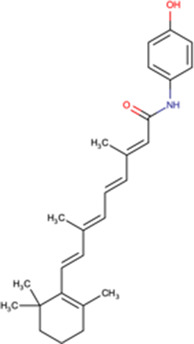	Inhibits interaction *in vitro* of DENV1 NS5:Impα/β (2.3 μM) [82]; DENV2 NS5:Impα/β (0.8 μM) [15]; DENV2 NS5:ImpαΔIBB (1.1 μM) [15]; ZIKV NS5:Impα/β (1.1 μM) [87] Inhibition of DENV2 NS5 nuclear accumulation in infected cells [15,47,77] Destabilises DENV 3 NS5 RdRp (thermostability assay) [44,47] 15 μM inhibits nuclear accumulation of influenza vRNPs (IF) [11]	Flaviviruses DENV1 (EDEN1)	EC50 = 2.6 μM (PFU, Huh-7) [15] EC50 (ADE^7^) = 0.8 μM (PFU, THP-1) [15] EC50 (ADE^7^) = 0.8 μM (PFU, PBMC) [15]
		DENV2 (EDEN2)	EC50 = 2.1 μM (PFU, Huh-7) [15]
		DENV2 (NGC)	EC90 = 2.0 μM (PFU, Vero) [77]
		DENV3 (EDEN3)	EC50 = 1.4 μM (PFU, Huh-7) [15]
		DENV4 (EDEN4)	EC50 = 2.1 μM (PFU, Huh-7) [15]
		ZIKV (Asian/Cook Islands/2014)	EC50 = 2.6 μM (PFU, Vero) [87]
		ZIKV (Asian/PF13-251013-1)	EC90 = 1 μM (PFU, multiple hosts) [78]
		ZIKV (African/ MR-766)	10 μM > 1000-fold (PFU, Vero) [78]
		WNV (Kunjin)	10 μM > 7-fold (PFU, Vero) [15] 10 μM > 100-fold (PFU, Vero) [77] 10 μM > 10-fold (PFU, Vero) [49]
		YFV	3 μM > 100-fold (BHK-21, PFU) [46]
		Alphavirus CHIKV	EC50 = 0.5/3.1 μM (PFU, multiple hosts) [46]
		Modoc	10 μM >100-fold (PFU, Vero) [77]
		Influenza VLPs^8^ (avian influenza A/MxA escape mutants)	10 μM > 4-fold inhibition (luciferase) [11]

1 Approved for human use in medical termination of pregnancy, hyperglycaemia treatment in Cushing's syndrome patients;

2 Analogue of mifepristone ((8S,13S,14S,17S)-17-hydroxy-13-methyl-11-phenyl-17-((trimethylsilyl) ethynyl)-1,2,6,7,8,11,12,13, 14,15,16,17-dodecahydro-3H-cyclopenta[a] phenanthren-3-one)) lacking progesterone receptor antagonism [52];

3 Approved for asthma, chronic obstructive pulmonary disease, allergic rhinitis and nasal polyps, inflammatory bowel diseases;

4 Approved for the treatment of asthma, allergic rhinitis;

5 Selectivity index > 9.3;

6 Progressed to clinical trials (incl. Phase III) for indications including breast, bladder and paediatric cancers, macular degeneration; other actions include increased phosphorylation of eIF2α, promoting an antiviral state not related to long-chain ceramide biosynthesis, PERK pathway or ATF-4 [15,55,77];

7 ADE model uses subneutralising concentrations of anti-E protein antibody prior to infection;

8 Mechanism of action against influenza to be determined.

**Abbreviations** (see Table 1): ADE, antibody-dependent enhancement of infection; DENV, Dengue virus; CFI, cell-based flavivirus infection assay (immunostaining for virus); CoIP, co-immunoprecipitation; CP, capsid protein; CPE, cytopathogenic effect (host cell); CHIKV, Chikungunya virus; EC50, half-maximal effective concentration; Est., estimated; FRAP, fluorescence recovery after photobleaching; FRET, fluorescence resonance energy transfer; GFP, green fluorescent protein; HAdV, human adenovirus; hCMV, human cytomegalovirus; HCV, hepatitis C virus; HIV, human immunodeficiency virus; HSQ NMR, heteronuclear single quantum coherence nuclear magnetic resonance; IBB, importin β-binding domain; IC50, half-maximal inhibitory concentration; IF, immunofluorescence; IN, integrase; lum, luminescence assay; NS5, non-structural protein 5; PBMC, peripheral blood mononuclear cell; PCR, polymerase chain reaction; PIC, preintegration complex; PFU, plaque forming units (infectious virus); PRV, pseudorabies virus; RdRp, RNA-dependent RNA polymerase; SV40, Simian virus 40; T-ag, SV40 large T-antigen; TCID, tissue culture infectious dose (estimation of viral load based on CPE); VEEV, Venezuelan equine encephalitis virus; VLP, virus-like particle; vRNP, viral ribonucleoprotein complex; VSV-G, vesicular stomatitis virus glycoprotein; WNV, West Nile virus; YFV, Yellow Fever virus; ZIKV, Zika virus.

The first indication that ivermectin might have a role impacting viral protein nuclear import, and thereby potential as an antiviral, was afforded in 2011 in a chemical compound library HTS to identify inhibitors of binding of IMPα/β1 to HIV-1 IN [14]. A nested counterscreening strategy confirmed ivermectin to be an agent likely directly interacting with IMPα/β1 rather than IN to inhibit IMPα/β1:IN binding (IC50 of ∼ 5 μM) [14]. Consistent with this, ivermectin was found to inhibit nuclear accumulation of IN and the well-characterised IMPα/β1-recognised nuclear import cargo SV40 large tumour antigen (T-Ag), but not of cargoes that are transported through IMP-dependent pathways that do not rely on IMPα, such as the IMPβ1-recognised telomere repeat factor-1 [14]. Ivermectin has since been shown to inhibit the binding of IMPα/β to a range of viral proteins, including DENV NS5 (see Figure 1), as well as host cell proteins (see Table 1).

Recent work has confirmed that the mechanism of action of ivermectin in this context is through direct binding to IMPα, resulting in structural changes that decrease α-helicity and likely impact flexibility, as implied by circular dichroism (CD)/thermostability (TSA) measurements (see Figure 2) [44]. These changes prevent interaction with NLS-containing viral/host protein cargoes, as well as IMPβ1; ivermectin can also dissociate preformed IMPα/β heterodimer, which is the form IMPα needs to be in to mediate nuclear import [44]. Thus, ivermectin prevents recognition of nuclear import cargoes by IMPα and the IMPα/β1 (see Figure 1C), significantly reducing their nuclear accumulation and thereby diminishing suppression of the host antiviral response in infection. Inhibition of nuclear accumulation by ivermectin has been demonstrated for many different viral proteins [14,18] as well as host proteins such as NF-kB p65 [45] in transfected and infected cell systems (see Table 1). Ivermectin's ability to inhibit the binding of IMPα to the viral proteins has also been shown in a cellular context using the technique of biomolecular fluorescence complementation [32].

The IMPα-dependent host-targeted mode of action of ivermectin explains the wide range of viruses for which ivermectin has demonstrated antiviral effects both *in vitro* and *in vivo* [18]. *In vitro* antiviral properties have been demonstrated for a wide range of viruses, including Betacoronavirus SARS-CoV-2 (COVID19), the lentivirus HIV-1, alphainfluenzavirus (influenza A), flaviviruses (DENV, WNV, ZIKV, yellow fever virus — YFV), alphaviruses (CHIKV, Sindbis, Semliki forest virus, VEEV), the Hendra henipavirus, mastadenoviruses (human adenovirus — HAdV — B and C), the BK betapolyomavirus and PSV varicellovirus (Table 1). In the case of DENV and ZIKV, ivermectin antiviral activity has been confirmed in an *ex vivo* human disease-relevant model of peripheral mononuclear blood monocytes (PBMCs) [15,44]. It is noteworthy that ivermectin was also independently identified by HTS for antiviral agents able to inhibit CHIKV replication using a luciferase reporter-carrying virus [46].

### Other inhibitors targeting IMPα/β1

Compound library HTS has enabled the identification of small molecule IMPα-targeting inhibitors other than ivermectin (see Table 1**)**. GW5074 was identified as an inhibitor of IMPα/β1-DENV NS5 binding [15,47]. Recent efforts to characterise the properties of GW5074 have revealed that, in analogous fashion to ivermectin, it binds to IMPα, eliciting changes in α-helicity/flexibility implicated by CD/TSA measurements (see Figure 2), that lead to dissociation of the IMPα/β1 heterodimer/inhibition of NS5 nuclear import [47]. This is likely the basis of GW5074's ability to limit infectious virus production of DENV, ZIKV, and WNV [47]. Another host-targeted inhibitor, gossypol (GSP) discovered in the same HTS that identified ivermectin (see above) [14] has been shown to inhibit interaction of IMPα/β1:Hendra virus (HENV) V protein [48]. GSP has also been shown to have antiviral effects against WNV [49].

*In silico* structure-based-drug-design HTS has also been successfully applied to identify nuclear transport inhibitors [17]. *In silico* screening of 1.5 million compounds for the ability to dock into the NLS-binding site of IMPα and thereby mimic binding of the VEEV CP NLS to IMPα identified a range of different small molecules that were subsequently confirmed experimentally as being able to inhibit binding of IMPα/β1 to VEEV CP. Several of the lead compounds, including 1111864 (see Table 1) were confirmed to inhibit CP nuclear accumulation, as well as VEEV replication in infected cells [17].

### Nuclear import inhibitors targeting specific viral proteins

In addition to the host-targeted inhibitors discussed above, several inhibitors have been identified that specifically target the virus side of the IMPα/β1-virus axis to prevent viral protein nuclear import (see Table 2). The steroid progesterone/glucocorticoid receptor antagonist mifepristone was the first such inhibitor to be identified in HTS, through its ability to inhibit the interaction of recombinant IMPα/β1 with HIV-1 IN [14]. Nested counterscreening was used to establish that its action was specific to IMPα/β1: IN and not due to direct effects on IMPα/β1 [14]), with more recent NMR studies confirming its direct binding to the IN core domain, with effects within the vicinity of key residues within the IN NLS [37]. Importantly, mifepristone was able to inhibit nuclear import of IN in an *in vitro* reconstituted nuclear import assay [37]. *In vitro* results had previously indicated strong antiviral activity against HIV-1 [50], with activity also documented against VEEV and HAdV [51–53]; since neither of these viruses possess an IN-homologue, the precise mechanism by which mifepristone impacts nuclear accumulation of CP in VEEV-infected cells [51] and of the HAdV genome in HAdV-infected cells [53] is unclear. A limitation on the clinical utility of mifepristone is the fact that it is abortifacient; medicinal chemistry and SAR analysis were able to be used to generate novel mifepristone analogues that lacked detectable progesterone receptor antagonism, but retained the ability both to inhibit VEEV CP nuclear accumulation and to limit VEEV infection [52], underlining how medicinal chemistry/SAR analysis can produce an inhibitor with improved properties.

The glucocorticoid budesonide was identified in the same HTS campaign as mifepristone (IC50 of c. 1 μM), subsequent characterisation using NMR confirming that budesonide also binds to the HIV-1 IN NLS region [37]. SAR/NMR analysis of closely related molecules showed that flunisolide also bound IN, but causes greater structural perturbation than budesonide, consistent with flunisolide being a more potent inhibitor of IN nuclear accumulation *in vitro* [37]. Importantly, both flunisolide and budesonide could be confirmed to inhibit nuclear import of the HIV PIC, as inferred from quantitative polymerase chain reaction analysis of unintegrated nuclear viral DNA [25]. Both budesonide and flunisolide also showed robust antiviral activity towards HIV-1, including in a single round replication assay (see Table 2**)**.

From HTS of *a* > 14 000 compound library for inhibitors of IMPα/β1:VEEV CP binding, followed by a nested counterscreen, many chemical scaffolds were identified that retained potent inhibitory activity [16]. Subsequent *in silico*-informed SAR analysis focussed on several of these, with compound G281–1564 confirmed to be a specific inhibitor of IMPα/β1:CP interaction. Experiments in transfected cells confirmed inhibition of CP but not SV-40T-Ag nuclear accumulation, with fluorescence recovery after photobleaching experiments demonstrating inhibitory effects on both the rate and extent of CP nuclear import. Importantly, G281–1564 was found to limit VEEV infection in cell culture (EC50 of 10.8 μM) [16]; subsequent work indicated that G281–1564 prevents delays in cell cycle progression, which in part may depend on CP nuclear localisation/ IMPα/β1:CP binding [54].

One of the most promising inhibitors targeting the virus side of the IMPα/β1-virus axis is N-(4-hydroxyphenyl) retinamide (4-HPR, a.k.a. fenretinide), which was identified through HTS as a specific inhibitor of IMPα/β1:DENV NS5 interaction (IC50 of 0.8 μM) [15]. 4-HPR was shown to reduce the nuclear accumulation of DENV NS5 in infected cells, as well as strongly inhibiting viral replication of all four circulating DENV strains (DENV1-4) with EC50 values between ∼1–2 μM, including in an *ex vivo* model of severe antibody-dependent enhanced (ADE) human infection using PBMCs [15]. 4-HPR also limits infection by other flaviviruses, including ZIKV (EC50 of 1–2 μM), WNV, and YFV (see Table 2). SAR analysis together with the use of other inhibitors confirmed 4-HPR's specificity, and that its mechanism of antiviral action is though inhibiting NS5 nuclear import, rather than other effects (e.g. on ceramide metabolism) [15]. TSA assays confirmed that 4-HPR destabilises DENV2 NS5 RNA-dependent RNA polymerase (RdRp) domain, and not IMPα [44], highlighting its specificity for the viral component of the host–virus interface.

It is of interest that 4-HPR has also been identified in HTS (see above) as an inhibitor of CHIKV [46], and reported to limit infection by the arbovirus Modoc and influenza A viruses. Like CHIKV, Modoc, and influenza virus do not have a correlate of flavivirus NS5 (well-conserved in DENV1-4, ZIKV, YFV, WNV, Japanese Encephalitis Virus) that localises in the nucleus of the infected host cell to impact transcription/splicing, so that 4-HPR's antiviral action in this case may be through other cellular pathways (ceramide biosynthesis/unfolded protein response) [47,55].

### Pre-clinical/clinical studies for IMPα targeting inhibitors

Host-directed agents that impact essential cellular activities like nuclear transport must be used with caution in a clinical setting. Of the small molecule inhibitor drugs targeted against the host IMPα/β1 side of the IMPα/β1:virus interface, ivermectin is the only one that has progressed from to human clinical trials for viral indications. Although it has an established safety profile in humans [43,56], and is FDA-approved for many parasitic infections [56–58], ivermectin targets host nuclear import function that is unquestionably important in the antiviral response, with titration of a large proportion of the IMPα repertoire of a cell/tissue/organ is likely to lead to toxicity. Where a host-directed agent like ivermectin can optimally be used to treat viral infection may well be in the initial stages of infection or potentially prophylactically by keeping the viral load low so that the body's immune system has an opportunity to mount a full antiviral response [44,59].

Pre-clinical studies in a lethal mouse PRV challenge study showed that ivermectin administration (0.2 mg/kg) 12 h post-infection rescued 50% of mice, while the same dose at the time of infection rescued 60% of mice [60]. Results from a human Phase III clinical trial for the treatment of patients presenting with fever and confirmed DENV diagnosis showed that daily administration (0.4 mg/kg for 3 days) was safe and effective at improving virus clearance, but the authors concluded that dosing regimen modification was required to achieve the clinical benefit, potentially due to the timing of administration of the doses [61].

The current SARS-CoV-2 pandemic has already seen >67 million infections and >1.5 million deaths (nearing 300 000 in the US alone [62]) worldwide [63]. SARS-CoV-2 is a respiratory virus, so it is noteworthy that pharmacokinetic modelling, based on both the levels of ivermectin achievable in human serum from standard 0.2 mg/kg dosing and robust measurement in large animal experiments, indicates that concentrations of ivermectin 10 times higher than the c. 2.5 μM EC_50_ indicated by *in vitro* experiments (Table 1) are likely achievable in the human lung [64]. Modelling based on different assumptions predicts lower values, but highlights the long-term stability of ivermectin in the lung (>30 days) [65]. Currently, almost 80 clinical trials are in progress globally for the treatment or prevention of SARS-CoV-2 [66–68], including variations on dosing regimens, combination therapies (see also [69–71]) and prophylactic protocols. Preliminary results from prophylactic study NCT04422561 of asymptomatic family close-contacts of confirmed COVID-19 patients indicate that two doses of ivermectin 72 h apart (0.25 mg/kg) are efficacious, resulting in an 87% relative risk reduction in the development of symptoms (58.4% of the untreated group vs 7.4% of the ivermectin-treated group).

Results from retrospective/observational trials for ivermectin [72–75] are also consistent with clinical benefit in the context of SARS-CoV-2 infection. Mymensingh Medical College Hospital (Bangladesh) reported that none of 115 subjects receiving a single 12 mg dose of ivermectin developed pneumonia/cardiovascular complications, compared with 9.8% (pneumonia) and 1.5% (ischemic stroke) in 133 control subjects [72]. Ivermectin-treated patients became SARS-CoV-2 negative faster (median 4 compared with 15 days), had shorter hospital stays (median 9 versus 15 days), and lower mortality (0.9 versus 6.8%). Importantly, significantly fewer ivermectin group patients developed respiratory distress (2.6 versus 15.8%), or required oxygen (9.6 versus 45.9%) or intensive care management (0.09 versus 8.3%). Comparable results were obtained in a 196 patient propensity-matched cohort study at Broward Health Medical Centre (Florida, U.S.A.) [73], with significantly lower mortality (13.3%) in subjects receiving ivermectin (0.2 mg/kg), compared with 24.5% mortality in those not receiving it, with more significant differences for patients with severe pulmonary involvement (mortality rates of 38.8 versus 80.7%). These early results are consistent with efficacy, but it is clear that only the results from large rigorous randomised clinical trials (RCTs) [66,67] will establish ivermectin's utility to treat or prevent SARS-CoV-2 infection.

### Pre-clinical/clinical studies for viral protein targeting inhibitors

Of the small molecule inhibitors targeting the virus side of the IMPα/β1:virus interface, only mifepristone has thus far been investigated clinically in any detail. Mifepristone was shown to reduce virus production and viral DNA copy number in HAdV-infected mice, concomitant with inhibition of HAdV genome nuclear import [53]. However, although a Phase I/II clinical trial of mifepristone (75–225 mg/day) in HIV patients indicated it was safe and well-tolerated, there was no significant effect on plasma HIV-1 RNA levels or CD4^+^ lymphocyte counts [76].

In the case of 4-HPR as a therapeutic for flavivirus infection, results are thus far restricted to mouse models. In AG129 mice, twice-daily dosage of 4-HPR at 180 mg/kg (resulting in ∼15 μM plasma concentration of 4-HPR), reduced viremia >50-fold in DENV2 infection [77]. In a lethal challenge model of ADE-DENV1 infection in AG129 mice, 20 mg/kg 4-HPR once-daily rescued 20% of mice, whilst twice-daily treatment rescued 70% of mice [15], concomitant with reduced inflammatory cytokine responses (tumour necrosis factor α, IL-6, -10, and -12) [15]. Efficacy was also seen in AG129 mice for ZIKV infection, where 4-HPR at 60 mg/kg twice-daily reduced viremia by ∼10-fold [78]. Previous human Phase II/III trials for a variety of adult and paediatric solid tumour indications have demonstrated 4-HPR plasma trough concentrations exceeding 7 μM (up to a peak of >50 μM) are achievable with a tolerable safety profile [79,80], which compares favourably with EC50 values of 1–2 μM for DENV in human PBMCs [15]. Based on these results as well as 4-HPR's established human safety and pharmacokinetic profiles in adults and children, human clinical trials are planned to investigate 4-HPR's exciting potential as a therapeutic and potentially prophylactic agent against DENV.

## Future prospects

It is clear from the last decade that small molecules targeting the host IMPα/β1:virus interface can be found that have potent antiviral activity, and exciting potential to treat human infection [66]. While host-targeted ‘broad spectrum’ inhibitors such as ivermectin can affect a range of viruses that share the need for the same nuclear import pathways as part of their lifecycle, potential toxicity must be considered, due to the essential nature of these import pathways for host cell function [4]. Virus-targeted inhibitors like 4-HPR, on the other hand, do not inhibit host protein nuclear import and hence are not toxic, but inevitably have a more restricted spectrum of antiviral activity as a result [66].

Conventional HTS (e.g. [14–16]) have proven effective at identifying promising lead compounds which are specific and effective at achievable concentrations, with *in silico* HTS emerging as a complementary approach to identify promising leads, and facilitate focussed library selection (e.g. [17,52]). Drugs such as ivermectin and 4-HPR are clearly exciting prospects, but the possibilities have by no means been yet exhausted in terms of agents that target the host IMPα/β1:virus axis, let alone other host IMP:virus interfaces. It is clear that further HTS and development of novel approaches to target these and other interfaces are likely to prove beneficial in the current landscape, in terms of identifying new possible agents to control existing and emerging viral pathogens.

## Perspectives

- Nuclear import is essential to the infectious cycles of a range of viruses of medical significance. Inhibitors of nuclear import targeting either the host or virus side of the IMPα/β1:virus interface have been identified in recent years, and shown to possess potent antiviral activity.
- Host-targeted inhibitor of nuclear import ivermectin is showing exciting early results as a treatment for COVID-19 infection. Virus-targeted inhibitor of nuclear import of flavivirus NS5, 4-HPR, appears to be an exciting prospect for future clinical trials.
- The process of nuclear import, and the IMPα/β1:virus interface in particular, remains a viable target of interest for the future identification and development of antivirals to tackle existing and emerging viral disease.

## Open Access

Open access for this article was enabled by the participation of Monash University in an all-inclusive *Read & Publish* pilot with Portland Press and the Biochemical Society under a transformative agreement with CAUL.

## Abbreviations

ADE: antibody-dependent enhanced
ARM: α-helical armadillo
CD: circular dichroism
CE: chicken embryo
DENV: dengue
HIV-1: human immunodeficiency virus-1
HTS: high-throughput screening
IBB: importin-β-binding
NLSs: nuclear localisation signals
NP: nucleoprotein
PBMCs: peripheral mononuclear blood monocytes
PIC: preintegration complex
RCTs: randomised clinical trials
SARS: severe acute respiratory syndrome
STATs: signal transducer and activators of transcription
TSA: thermostability
WNV: West Nile virus
ZIKV: Zika
